# Lower Limb Exoskeleton Gait Planning Based on Crutch and Human-Machine Foot Combined Center of Pressure

**DOI:** 10.3390/s20247216

**Published:** 2020-12-16

**Authors:** Wei Yang, Jiyu Zhang, Sheng Zhang, Canjun Yang

**Affiliations:** 1 Ningbo Research Institute, Zhejiang University, Ningbo 315100, China; simpleway@zju.edu.cn (W.Y.); ycj@zju.edu.cn (C.Y.); 2 School of Mechanical Engineering, Zhejiang University, Hangzhou 310027, China; 3 School of Instrumentation Science and Engineering, Harbin Institute of Technology, Harbin 150001, China; 19b901013@stu.hit.edu.cn

**Keywords:** gait planning, stride length, center of pressure, human–machine interaction

## Abstract

With the help of wearable robotics, the lower limb exoskeleton becomes a promising solution for spinal cord injury (SCI) patients to recover lower body locomotion ability. However, fewer exoskeleton gait planning methods can meet the needs of patient in real time, e.g., stride length or step width, etc., which may lead to human-machine incoordination, limit comfort, and increase the risk of falling. This work presents a human-exoskeleton-crutch system with the center of pressure (CoP)-based gait planning method to enable the balance control during the exoskeleton-assisted walking with crutches. The CoP generated by crutches and human-machine feet makes it possible to obtain the overall stability conditions of the system in the process of exoskeleton-assisted quasi-static walking, and therefore, to determine the next stride length and ensure the balance of the next step. Thus, the exoskeleton gait is planned with the guidance of stride length. It is worth emphasizing that the nominal reference gait is adopted as a reference to ensure that the trajectory of the swing ankle mimics the reference one well. This gait planning method enables the patient to adaptively interact with the exoskeleton gait. The online gait planning walking tests with five healthy volunteers proved the method’s feasibility. Experimental results indicate that the algorithm can deal with the sensed signals and plan the landing point of the swing leg to ensure balanced and smooth walking. The results suggest that the method is an effective means to improve human–machine interaction. Additionally, it is meaningful for the further training of independent walking stability control in exoskeletons for SCI patients with less assistance of crutches.

## 1. Introduction

More than 250,000 individuals annually sustain spinal cord injuries worldwide, mainly due to traffic accidents and fall from heights [1]. Spinal cord injury (SCI) patients can become paraplegic due to lesion characteristics. Moreover, long-term sitting and lying may cause poor health conditions and complications, e.g., muscular atrophy, pressure sores, constipation, and osteoporosis. Therefore, helping SCI patients to stand, walk, and engage in self-care may mitigate a crucial social problem.

With the development of wearable robotics and sensors, increased attention has been paid to the research of the lower-limb exoskeleton for the locomotion impaired, such as SCI and stroke patients. Zhang et al., Kawamoto et al., and Nilsson et al. developed exoskeleton systems for stroke rehabilitation through referencing normal walking gait guidance or reference gait-based impedance control [2,3,4]. Husain et al., Fineberg et al., and Jung et al. are more concerned about various functions of the exoskeleton to help SCI patients walk independently [5,6,7]. The former series focused on topics, such as patient walking intention recognition and joint torque control, and the latter on issues, such as human-machine dynamic balance control, walking mode switch for various terrains, and gait trajectory planning. Still, a significant amount of research and development is required to assist SCI patients to walk naturally and in balance, especially regarding exoskeleton gait planning. Besides efficacy, lack of community involvement, etc., the performance of self-controlled walking balance inhibits the wide application of commercial medical lower-limb exoskeletons on the market, such as Rewalk [8], HAL [9], and Ekso [10].

The simplest method to perform a “standard” gait is to predefine the normal gait trajectory. The desired joint trajectory is either recorded from several healthy volunteers or extracted from a gait analysis database. To improve the adaptation to various subject heights, the predefined gait trajectory is usually parameterized by lower limb sizes. Suzuki et al. prerecorded hip and knee joint angles from a healthy subject and divided a gait cycle into stance and swing phases, which are triggered by plantar reaction forces and torso tilt angles [11]. Similarly, Mina is controlled by a predefined gait trajectory [12], and gait-related parameters, including single- or continuous-step mode selection, walking speed, and step transition duration, can be tuned. Wang et al. proposed the MINDWALKER exoskeleton for gait assistance in the coronal and sagittal planes [13]. The gait is prerecorded by a healthy subject walking in MINDWALKER in zero-assistance mode. The predefined standard gait in the sagittal plane guarantees a natural walking posture, and step width control realized by online adjustment of exoskeleton hip abduction/adduction joints in the coronal plane ensures walking stability. Therefore, gait planning online or offline with additional measured information has been studied in recent years. Jeon et al. presented a fast wearable sensor-based gait phase classification method with the help of a convolutional neural network, which represents human-machine walking intention and is useful for exoskeleton motion control [14]. The polymer optical fiber sensors reported by Leal-Junior et al. show promising application scenarios for soft and wearable gait measurement for exoskeleton online gait planning [15].

Jung et al. presented the online computation of centroidal momentum (CM) [16], i.e., linear and angular momenta at the center of mass (CoM), in the exoskeleton-supported walking, which is regarded as a stability index to estimate the actual state of balance. Preliminary trials confirmed the assumption, and further research is in the progress of using CM to trigger a controller of the exoskeleton to maintain or recover the balance. Aphiratsakun et al. proposed a leg exoskeleton balancing control using a zero-moment point (ZMP) and a fuzzy logic controller [17]. The ground contact points on each foot were measured by a load cell and compared with the target ZMP, and input the differences into the controller, which generates the compensating angles of the left and right (L/R) ankle joints to position ZMP in the convex hull of the support area. Similarly, the center of pressure (CoP) of a human-machine system was investigated by Kim et al. for walking balance validation [18]. With measurements of both human gravity and exoskeleton support force, the CoP is calculated, and the stability condition is judged. Chen et al. calculated the CoP of a human-machine-crutch system and controlled an exoskeleton with an offline designed gait [19]. During walking, the gait is modified online if the calculated CoP exceeds a predefined stable area. Deng et al. used the capture point theory for biped robot balance control to guide the target landing point of a human and exoskeleton swing foot [20]. The instantaneous capture point is obtained by modeling the human-machine system, and the gait trajectory is corrected to solve the instability problem caused by random forward/backward leaning of the subject’s upper body. Most of the above work is focused on human-machine dynamic stability during walking with the help of gait planning. Although CoP, ZMP, CoM, etc. are key facts to guide gait planning, the influence of crutches is not always considered. The subject’s strength on crutches somehow determines the human-machine walking balance state. We focus on the support distribution of crutches and exoskeleton soles, to guide gait planning. This paper designs a gait planning algorithm, aiming to improve human-machine coordination and gradually improve a subject’s active stability control during walking. The gait planning uses CoP calculation based on crutch reaction force and human-machine plantar force, and stride length mapping determined by the calculated combined CoP.

The remainder of this paper is organized as follows. Section 2 introduces the exoskeleton prototype, mathematical method, and related simulation. Section 3 presents the experimental design for gait planning algorithm validation. Results are analyzed and the effects of the algorithm are discussed in Section 4. Section 5 relates our conclusions, along with future research directions.

## 2. Materials and Methods

**Figure 1:**
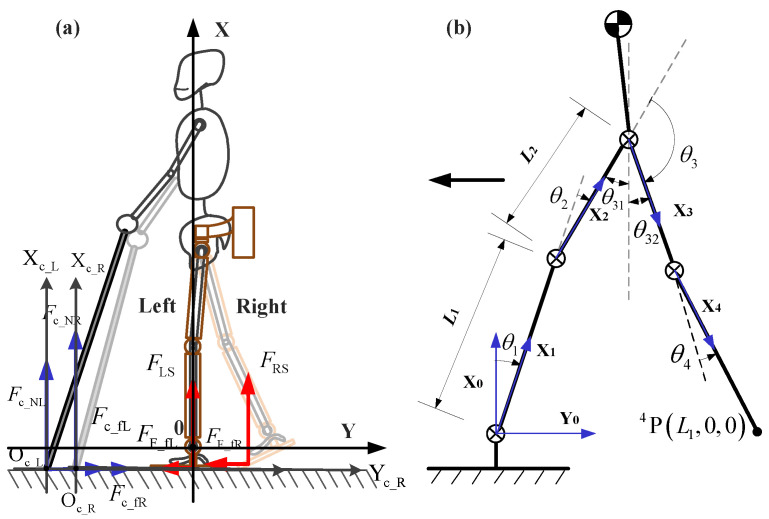
Human-exoskeleton-crutch system in the sagittal plane. (**a**) The local coordinate system is located at the rotation center of support of the leg ankle joint. The L/R crutch coordinate is located at the endpoint of the crutch. (**b**) Four-DoF kinematic model of the lower-limb exoskeleton. Denavit-Hartenberg parameters are tagged, including axes on joints and rotation angles of adjacent links.

In this work, a commercial lower-limb exoskeleton (UGO, RoboCT, Inc., Hangzhou, China) is adopted as a testbed for gait planning algorithm validation. Besides, customized crutches and exoskeleton soles are designed as the accessory equipment for the crutch endpoint pressure and human-machine foot pressure measurement, respectively. Thus, the combined CoP can be calculated based on the pressure of the crutch endpoint and human-machine foot. By establishing the relationship between stride length and combined CoP, the stride length can be obtained. Finally, due to the determination of the stride length, the target gait will be planned by forward and inverse kinematic models. For the forward and inverse kinematics models’ validation, the gait planning algorithm is simulated by MATLAB (Mathworks). The balance control of the human-machine system in the sagittal plane is the primary concern, since the hip and knee flexion/extension (f/e) joints with the developed lower-limb exoskeleton are motor driven. The human-exoskeleton-crutch system in the sagittal plane is shown in Figure 1a. The local coordinate system is established, in which *X*- and *Y*-axes indicate the vertical and horizontal moving directions, respectively. The origin of the local coordinate system is located at the ankle joint of the exoskeleton support leg. Figure 1b shows the four-DoF kinematic model of the lower-limb exoskeleton. Since the torso motion does not affect forward/inverse kinematic modeling for gait planning, the torso is simplified as one rigid body.

### 2.1. Exoskeleton

**Figure 2:**
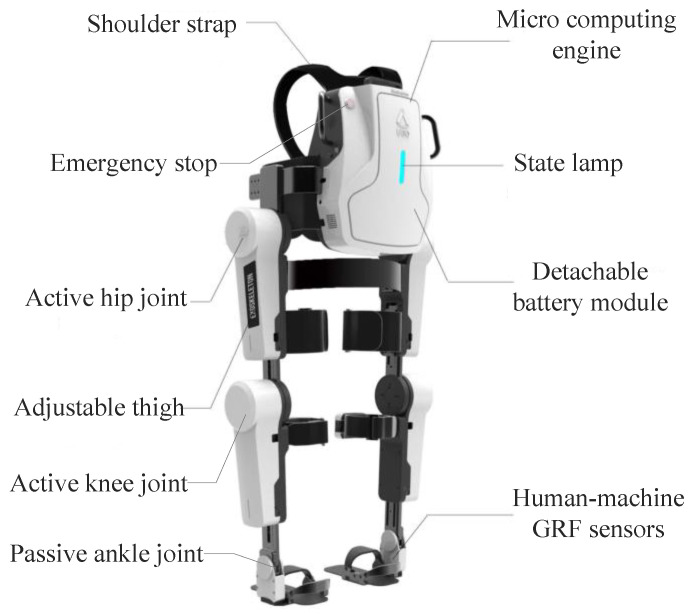
Lower limb exoskeleton system UGO developed by RoboCT, Inc.

The powered lower-limb exoskeleton system UGO (Figure 2) is developed to assist the balanced walk of patients with SCI or stroke. It is designed for subjects with heights between 1.5 and 1.9 m, and weigh below 100 kg. The hip and knee f/e joints are driven by servo motors and harmonic reducers coupled with a maximum rated torque of 100 Nm, while the ankle dorsi-flexion/ plantar-flexion joint is fully passive. All joints in the sagittal plane are limited to a certain range of motion, i.e., hip flexion (+110°) and extension (−40°), knee flexion (+95°) and extension (0°), ankle dorsi-flexion (+20°), and plantar-flexion (−30°). It should be emphasized that the passive ankle joint will not affect the gait planning since we focus on the ankle joint coordinates during forwarding/inversing kinematics instead of the footplate coordinate. Each exoskeleton motor-driven joint is equipped with a magnetic rotary encoder (AS5048A, AMS, Inc., Graz, Austria) with 14-bit resolution, and a torque sensor for joint angle and torque measurement, respectively. The torque sensor is customized with a range of 150 Nm, resolution of 0.05 Nm, and an accuracy of 0.3% FS. Both angle and torque signals are recorded with a sampling rate of 100 Hz.

### 2.2. Foot and Crutch Ground Reaction Force (GRF) Measurements

#### 2.2.1. Foot GRF Measurement

**Figure 3:**
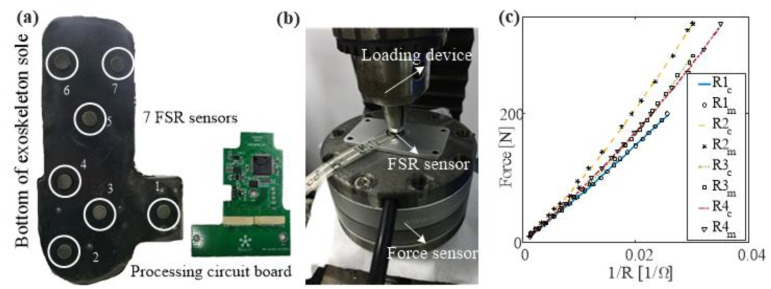
Calibration of accessory equipment. (**a**) Seven force-sensing resistor (FSR) sensors were mounted at the bottom of the exoskeleton sole to measure human-machine coupled ground reaction force (GRF). (**b**) Each FSR sensor was calibrated with a loading test bench by a force sensor (M4325K, Sun Rise Instrument, Inc., Nanning, China) with a range of 400 N and an accuracy of 0.5% FS. (**c**) Fitted results of calibration. Maximum RMS error of all calibration results was 3.601 N.

Force-sensing resistor (FSR) sensors mounted on exoskeleton soles and crutches determine the GRFs and the CoP of the human-exoskeleton-crutch system. Figure 3a shows a human-machine GRF measurement sole, including seven FSR sensors, a rubber baseboard, and a processing circuit. FSR sensors were calibrated first with a designed force loading test bench, as shown in Figure 3b. All results are fitted by fifth-order polynomials. The calibration results are shown in Figure 3c. The maximum RMS error of all calibration results is 3.601 N.

#### 2.2.2. Crutch GRF Measurement

**Figure 4:**
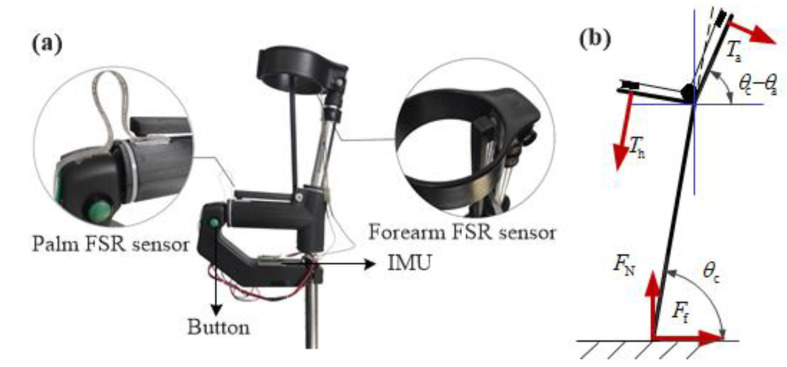
Customized crutch for GRF measurement. (**a**) Locations of palm and forearm FSR sensors, as well as the mounting position of inertia measurement unit (IMU) for crutch pitch angle measurement. (**b**) With force analysis, the crutch, normal and tangential components of GRF are obtained.

Chen et al. considered the influence of a crutch on human-machine balance control and designed crutches with FSR sensors at the bottom [19]. On the one hand, these sensors are helpful to measure GRF directly and conveniently. On the other hand, the interference caused by the change of the pitch angle of the crutch is an uncertain point in the application of GRF. To solve this problem, an indirect measurement of GRF for the crutch is proposed in this work. Since only the palm and forearm contact the crutch, FSR sensors are mounted on both contact areas, as shown in Figure 4a. One inertial measurement unit (IMU, JY901, Wit-Motion, Inc., Shenzhen, China) is mounted on the crutch to measure the crutch roll/pitch/yaw angles. With the above sensors, GRF on the crutch can be calculated by simple force analysis, as shown in Figure 4b. Compared to the direct measurement method in [19], this indirect measurement method is: (i) Easily acquires the contacting force data of the palm and forearm, and (ii) has the ability to remove the disturbance of the crutch pitch angle. The disadvantage is that some yaw, pitch, roll angle errors, palm, and forearm contacting force errors may lead to large vertical GRF errors. Therefore, calibration was performed in the following part. For this model, we assume the crutch endpoint is almost landed in the sagittal plane when the crutch yaw angle is within a range of −10~15 degree. Here, −10 degree denotes the inner side yaw angle boundary and 15 degree is the outside yaw angle boundary. $F_{N}$ and $F_{f}$ are the normal and tangential components, respectively, of GRF; $\theta_{c}$ and $\theta_{a}$ are the crutch pitch angle and intrinsic geometry angle, respectively; and $T_{h}$, $T_{a}$ are the palm and forearm pressure loaded on the crutch when the subject uses it for balance control:

(1) $$\left\{ \begin{array}{l} F_{N}=T_{a}\cos(\theta_{c}-\theta_{a})+T_{h}\sin\theta_{c}\\ F_{f}=-T_{a}\sin(\theta_{c}-\theta_{a})+T_{h}\cos\theta_{c} \end{array}\right..$$

**Figure 5:**
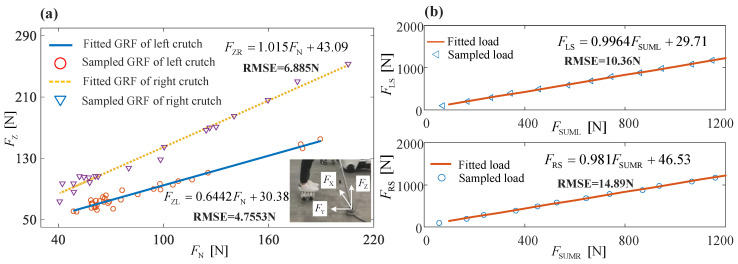
Calibration results of L/R crutches and L/R soles. (**a**) Calibration results of L/R crutches. $F_{\mathrm{ZL}}$ and $F_{\mathrm{ZR}}$ denote vertical components of L/R crutch endpoint GRF, respectively, and $F_{N}$ is the calculated vertical components of GRF based on measured palm and forearm pressure. (**b**) Calibration results of L/R soles. $F_{\mathrm{LS}}$ and $F_{\mathrm{RS}}$ are L/R gravities loaded on the soles, respectively. $F_{\mathrm{SUML}}$ and $F_{\mathrm{SUMR}}$ are the sum of gravities measured by 7 FSR sensors on L/R soles, respectively.

By using Equation (1), the normal component of GRF was calculated for each crutch, since both the palm and forearm pressures were measured as well as the pitch angles of crutches. To ensure accurate GRF measurement, further calibration was done with the commercial load cell. The real vertical component of GRF loaded by the crutch, denoted as $F_{Z}$, was measured by a load cell. The subject was asked to use the crutches to maintain balance while the upper body was leaning slightly forward. After data recording, the subject was asked to adjust his posture slightly and stand still to record data again. Figure 5a shows the calibration results of L/R crutches. Since the subject was accustomed to holding the crutches in a certain posture for balance control, most normal components of GRF were less than 100 N. Figure 5b is the calibration results of L/R soles. RMS errors of L/R soles are 10.36 and 14.89 N, respectively.

### 2.3. Combined CoP and Stride Length Calculation

The main idea of gait planning comes from the combined CoP based on the crutch GRF and human-machine GRF, which differs from the conventional CoP that is acquired from human plantar force interacting with the exoskeleton soleplate. We are concerned not only with subject gravity but with the GRF from the crutch, as well as that caused by the exoskeleton’s own gravity. With the force analysis shown in Figure 1, this combined CoP in the sagittal plane can be determined as:

(2) $$CoP_{y}=\frac{\sum_{i=1}^{4}\left(y_{i}\cdot F_{i}\right)}{\sum_{i=1}^{4}F_{i}},$$

where $CoP_{y}$ is the y-coordinate of the combined CoP in the local coordinate system. $y_{i}$(*i* = 1, 2, 3, 4) are the y-coordinates in the support ankle frame, i.e., X0-Y0 coordinate, as shown in Figure 1b, which denote the L/R crutch and L/R foot, respectively. $F_{i}$(*i* = 1, 2, 3, 4, which denote $F_{\mathrm{LS}}$, $F_{\mathrm{RS}}$, $F_{c\_\mathrm{NL}}$, $F_{c\_\mathrm{NR}}$) are the corresponding GRFs. With this in mind, the combined CoP can be obtained online as long as the crutch and foot coordinates and GRFs are determined.

**Figure 6:**
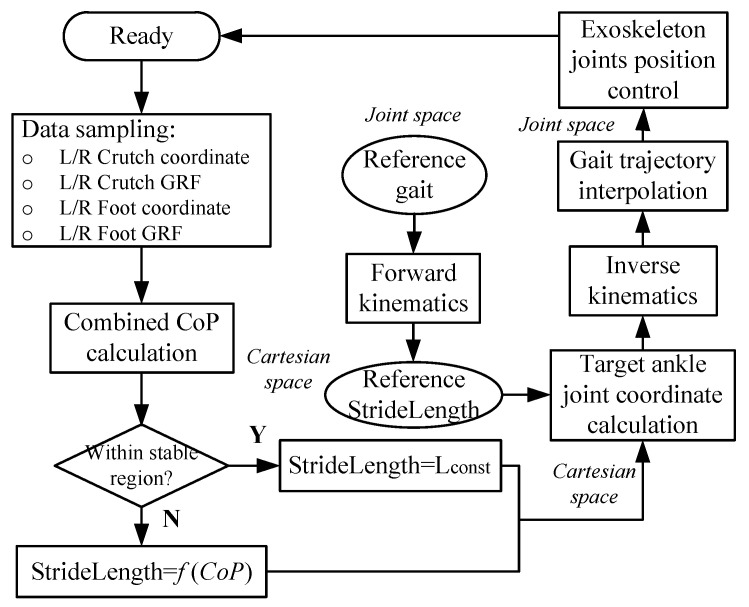
Algorithm for gait planning based on combined CoP. Once combined CoP is calculated, the stride length is obtained with a predefined mapping function. Compared with the reference stride length, the target gait trajectory is determined by converting the inverse kinematics. $L_{\mathrm{const}}$ is a constant stride length. When the combined CoP locates within a stable region (between L/R legs), the next stride length is determined to be $L_{\mathrm{const}}$.

Figure 6 shows the algorithm for gait planning based on combined CoP, which is obtained from the measured and calculated results: L/R crutch coordinates, L/R crutch GRF, L/R foot coordinates, and L/R foot GRFs. The L/R foot coordinates can be acquired directly by the four-DoF forward kinematics in support ankle coordinate (see Figure 1b), while the L/R crutch coordinates are calculated based on the forward kinematics of the support shank, thigh, torso, and upper limb-crutch serial-links model. The torso angle in the sagittal plane is measure by IMU placed on the torso. The upper limb-crutch coupled link pitch angle is measured by IMU mounted on the crutch. The stride length is determined by the combined CoP through a mapping function. The reference stride length is acquired from forward kinematics based on the reference gait. With the stride length and reference stride length, the target ankle joint coordinate can be calculated, and is converted into the joint space by inverse kinematics. After joint space gait sequence interpolation, the target gait trajectory is determined for the current gait semi-cycle. After gait planning before each swing, the human exoskeleton system swing step is triggered by button 2 and the planned semi-cycle gait will be performed during the swing phase. That is to say, Figure 6 denotes the procedure of each single swing step.

To establish a mapping relationship between the combined CoP and stride length, the subject’s walking behavior with the exoskeleton was studied. Based on walking tests with nominal reference gait (Kirtley, 2013) on healthy subjects and SCI patients with a height from 1.5 to 1.9 m, the stride length is mostly between 600 and 1200 mm, which coincides well with experimental data from Mendoza et al. [21]. Since a further relationship between the combined CoP and stride length is still unclear and may depend on human behavioral analysis, we simplified it to a linear mapping:

(3) $$L_{\mathrm{stride}}=\left\{ \begin{array}{l} a\cdot CoP_{y}+b\;-500\leq CoP_{y}\leq 0\\ L_{\mathrm{const}}\;\;\;\;\mathrm{else} \end{array}\right.,$$

where $L_{\mathrm{stride}}$ is the stride length, denoting the *Y*-axis length of the swing ankle joint from the beginning of swing to the termination of swing during one step, a and b are linear mapping function coefficients, and $L_{\mathrm{const}}$ is constant. With the condition that:

(1) The stride length of a subject walking in an exoskeleton is mostly between 600 to 1200 mm based on trials with nominal reference gait [22];
(2) The relationship between the combined CoP and the desired stride length is linear;
(3) $CoP_{y}$ is bounded within [−500, 0] mm when it is located in the negative *Y*-axis. We did a pre-test for a total of 100 steps on 5 healthy subjects, and the $CoP_{y}$ was always within [−500, 200] mm.

Thus, a = −1.2, b = 600. The constant $L_{\mathrm{const}}$ = 600 reflects that the stride length, when a subject walks in a stable region, is 600 mm.

### 2.4. Forward and Inverse Kinematic Modeling

Consider a simplified lower limb exoskeleton with four DoF, as shown in Figure 1b. Only pitch angles in the sagittal plane are of concern. Hip L/R f/e joints are considered to coincide in the sagittal plane. The ankle joint of the support leg is set as the origin of frame 0. The coordinate of the swing ankle joint in frame 4 is P4. With a forward kinematic model, we obtain its coordinate in the local coordinate system (frame 0). The Denavit–Hartenberg (DH) table of the Craig version [23] is shown in Table 1.

With the DH parameters from Table 1, the transformation matrix from the *i*-1th to *i*th coordinate can be obtained as:

(4) Tii−1=[cθi−sθi0ai−1sθicαi−1cθicαi−1−sαi−1−sαi−1disθisαi−1cθisαi−1cαi−1cαi−1di0001].

Thus, the transformation matrix from the fourth frame to the base frame can be represented as:

(5) T40=T10·T21·T32·T43.

The swing ankle joint coordinate is then calculated as:

(6) P0=T40·P4.

With the forward kinematic model, the swing ankle joint coordinate is obtained when the gait trajectory is known. We adopt the gait trajectory from Kirtley (2013) as the reference gait. Hence, the reference swing ankle joint coordinate in Cartesian space can be calculated, which is denoted as $\left(y_{a\_r},x_{a\_r}\right)$. Similarly, the reference hip joint coordinate in Cartesian space $\left(y_{h\_r},x_{h\_r}\right)$ is calculated. Since the target gait planning is a scaling operation based on the reference gait (the reference ankle joint and hip joint coordinates) in Cartesian space, the target swing ankle joint and hip joint coordinates are calculated by:

(7) $$k_{a}=\frac{L_{\mathrm{stride}}(k)+L_{\mathrm{stride}}(k-1)}{2L_{\mathrm{stride}\_r}}$$

(8) $$\left\{ \begin{array}{l} y_{a\_t}=k_{a}\cdot y_{a\_r}\\ x_{a\_t}=\lambda_{a}\cdot k_{a}\cdot x_{a\_r} \end{array}\right.$$

(9) $$\left\{ \begin{array}{l} y_{h\_t}=k_{a}\cdot y_{h\_r}\\ x_{h\_t}=\lambda_{h}\cdot k_{a}\cdot x_{h\_r} \end{array}\right.,$$

where $k_{a}$, $\lambda_{a}$, and $\lambda_{h}$ are the stride length scale, ankle, and hip optimization factors, respectively. The scale factor $k_{a}$ is determined by the target and reference stride lengths. The larger the target stride length is, the larger the scale factor $k_{a}$ will be, leading to magnified hip and ankle joint coordinates in Cartesian space compared to reference ones. Both $\lambda_{a}$ and $\lambda_{h}$ are within [0.9, 1.1] to prevent singular solutions. $L_{\mathrm{stride}}$ and $L_{\mathrm{stride}\_r}$ are the target and reference stride lengths, respectively. *k* denotes the *k*th semi-cycle of walking. $\left(y_{a\_t},x_{a\_t}\right)$ and $\left(y_{h\_t},x_{h\_t}\right)$ are the planned swing ankle joint and hip joint coordinates, respectively.

Once the target swing ankle joint and hip joint coordinates are obtained, gait planning becomes an inverse kinematic calculation. The geometric solution of the inverse kinematic of the above 4-DoF kinematic model is:

(10) $$\left\{ \begin{array}{l} \theta_{4}=a\cos\frac{L_{\mathrm{HA}\_\mathrm{SW}}^{2}-L_{1}^{2}-L_{2}^{2}}{2L_{1}L_{2}}\\ \theta_{2}=a\cos\frac{L_{\mathrm{HA}\_\mathrm{ST}}^{2}-L_{1}^{2}-L_{2}^{2}}{2L_{1}L_{2}}-180\\ L_{\mathrm{HA}\_\mathrm{SW}}^{2}={\left(y_{a\_t}-y_{h\_t}\right)}^{2}+{\left(x_{a\_t}-x_{h\_t}\right)}^{2}\\ L_{\mathrm{HA}\_\mathrm{SW}}^{2}=y_{h\_t}^{2}+x_{h\_t}^{2} \end{array}\right.$$

(11) $$\left\{ \begin{array}{l} \theta_{31}=90+\theta_{2}-\mathrm{atan}2(x_{h\_t}+\sqrt{L^{2}{}_{HA\_ST}-k_{2}^{2}},k_{2}+y_{h\_t})\\ k_{2}=L_{1}+L_{2}\cos\theta_{2} \end{array}\right..$$

(12) $$\left\{ \begin{array}{l} \theta_{32}=\gamma_{4}-\mathrm{atan}2(y_{a\_t}-y_{h\_t},x_{a\_t}-x_{h\_t})\\ \gamma_{4}=a\sin(\frac{L_{1}\sin\theta_{4}}{L_{\mathrm{HA}\_\mathrm{SW}}}) \end{array}\right..$$

(13) $$\theta_{1}=-\theta_{2}-\theta_{31},$$

where $L_{\mathrm{HA}\_\mathrm{SW}}$ and $L_{\mathrm{HA}\_\mathrm{ST}}$ are the respective distances from the hip joint to the swing and support ankle joints. Both $k_{2}$ and $\gamma_{4}$ are intermediate variables. $\theta_{31}$ and $\theta_{32}$ are hip joint angles for the support and swing leg, respectively. For exoskeleton hip and knee f/e joint control, the target gait is defined by:

(14) $$\theta =[\theta_{2},\theta_{31},\theta_{32},\theta_{4}].$$

### 2.5. Gait Planning Algorithm Simulation

**Figure 7:**
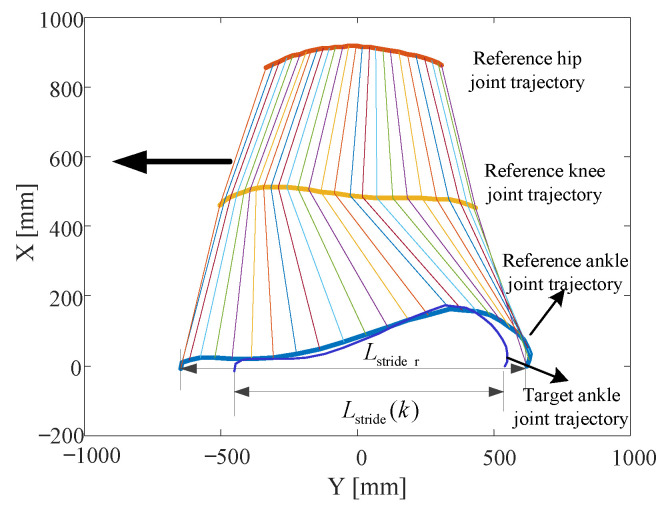
Resultant reference trajectories in Cartesian space and an example of a target ankle joint trajectory. Lower limb sizes are $L_{1}$ = 490 mm, $L_{2}$ = 430 mm, and $L_{stride\_r}$ = 1290.52 mm is the resultant stride length with the reference gait.

The reference Cartesian position coordinates of hip, knee, and ankle joints can be obtained through forward kinematics. Figure 7 shows the resultant reference trajectories in Cartesian space. An example of the target ankle joint trajectory is plotted for comparison. Having assumed $L_{\mathrm{stride}}(k)$ and $L_{\mathrm{stride}}(k-1)$, $k_{a}$ is obtained through Equation (6). Thus, the target ankle and hip joint trajectories are planned.

**Figure 8:**
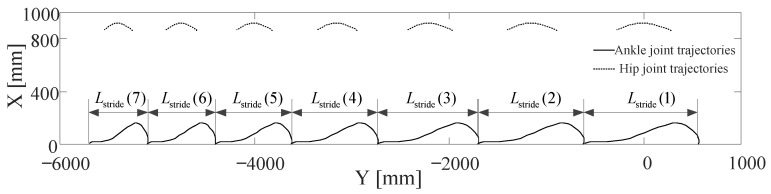
Left ankle joint and hip joint trajectories derived from planned gait with forward kinematics. Stride length can be obtained with simulation results, and the RMS error of seven left leg steps is 2.315 mm.

Since the lower limb exoskeleton is powered by motor-driven joints, including the hip and knee f/e, the planned Cartesian position coordinates of the ankle and hip joints should be converted to the joint space through inverse kinematics as shown in Equations (10)–(14). Consecutive steps of walking are simulated with a sequence of stride lengths, $L_{stride}$ = [1245, 1150, 1050, 950, 850, 750, 650]. Figure 8 shows the planned left ankle joint and hip joint trajectories derived from the planned gait. Simulated stride lengths are obtained by subtracting the first and last Y-coordinate of ankle joint trajectories during each step. Compared to the input stride lengths, the RMS error of seven left leg steps is 2.315 mm.

### 2.6. Online Gait Planning Walking

**Figure 9:**
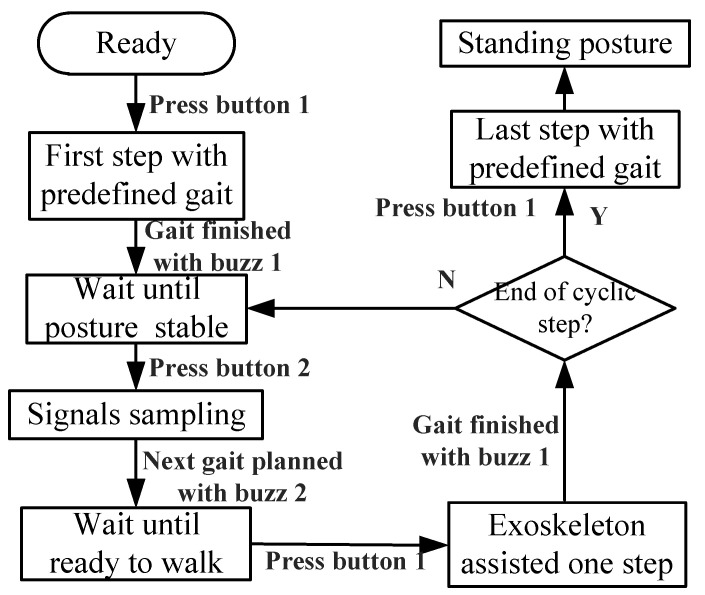
Exoskeleton-assisted walking test procedure. The subject was told to press buttons 1 and 2 before walking and signal sampling, respectively. The subject was reminded with buzz 1 that the current gait was finished, and with buzz 2 that signal sampling was completed. The gaits of the first and last steps were predefined and independent of CoP.

The procedures of exoskeleton-assisted walking are shown in Figure 9. For the first step, the gait was predefined to transfer from the standing posture to the double support phase with the left leg in front. At this moment, the subject would adjust L/R crutches to a certain posture to assure stability. Button 2 was pressed once the subject felt stable, and the corresponding signals were sampled to calculate the combined CoP and stride length, followed by gait planning in the micro-computing engine within several milliseconds. When the subject heard buzz 2, indicating the completion of the next step in gait planning, the subject would walk one step with an exoskeleton-assisted gait once button 1 was pressed. Buzz 1 reminded the subject when the current step was completed. The procedure continued until the end of the cyclic step. The walking test was ended when the last step was transferred from the double support phase to the standing posture.

## 3. Experiment

Three experiments were conducted to respectively verify (1) the advantage of selected linear mapping, (2) feasibility of the gait planning algorithm, and (3) how balanced walking is ensured. One healthy subject (male, 28 years of age, 70 kg, 1.76 m) volunteered for the second experiment, and 5 healthy volunteers (5 males, with 29.20 ± 3.90 (SD) year of age, 74.4 ± 4.39 (SD) kg, 1.74 ± 2.95 (SD) m) were recruited for the first and third experiments. Informed consent was obtained from the volunteers.

### 3.1. Stride Length Mapping Function Verification Test

The comparison tests were designed to prove the suitability of linear mapping for the subject to learn how to control the crutch landing point. Another two quadratic mapping functions (denoted as Quad1 and Quad2) were selected for comparison. $L_{stride}$ and $CoP_{y}$ of both quadratic functions were bounded with [600, 1200] and [−500, 200], respectively, which is the same as the linear function. Five volunteers were recruited and each one repeated 3 sets of walking trials with 27 steps, respectively. For the first set, a linear mapping function (denoted as Line1) was applied to determine stride length while for the other two sets, Quad1 and Quad2 were applied, respectively. The subjects were asked to control their weight distribution between the legs and crutches to realize the target stride length. For the first 9 steps, the target stride length was 650 mm. For the next 9 steps, the target stride length was 800 mm, and for the last 9 steps, the target stride length was 1000 mm. After each step, the realized stride length and deviation from the target one were told to the subject as feedback for adjustment of the next step. Deviations of the last 6 steps among every 9 steps were recorded.

### 3.2. Online Gait Planning Algorithm-Based Walking Test

**Figure 10:**
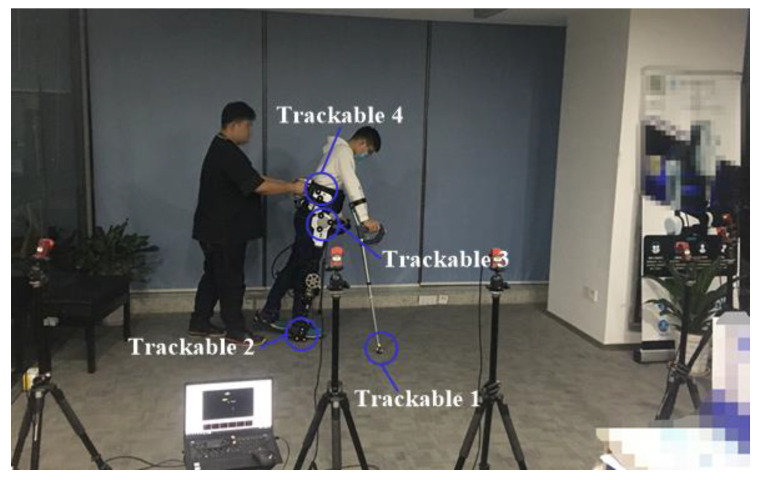
Exoskeleton-assisted walking test. Six cameras were grouped for motion capture of four trackable points. The exoskeleton was programmed to perform seven steps, including the first and last predefined transfer steps. The gaits of the other five steps were planned online based on CoP results.

The exoskeleton-assisted walking test was motion captured by an OptiTrack motion capture system (Natural Point, Inc., Corvallis, OR, USA) as shown in Figure 10. Four trackable points (each trackable combines three markers) were built for motion tracking of the right crutch endpoint, exoskeleton right ankle joint, hip joint, and trunk. With the first trackable, the landing coordinate and the pitch angle of the right crutch endpoint were recorded. With the other three trackable points, the right hip and knee f/e angles and trunk tilt angles were recorded. The right ankle joint coordinate was also recorded by trackable 2. The exoskeleton system stored the sampling signals and the calculation results, including L/R hip and knee angles, torques, GRFs from L/R human-machine soles, crutches, palm and forearm pressures loaded on L/R crutches, calculated CoP, and planned gait of each step. Six trials were conducted on one volunteer, and seven steps were performed for each trial because of the limited range of the motion capture system.

### 3.3. Balanced Walking Verification Test

The comparison tests were designed for the verification of balance control that was realized by online gait planning. The same 5 volunteers were recruited for exoskeleton-assisted walking tests. Each one was asked to finish 10 steps of walking for 2 trials: one trial was conducted with the aforementioned online gait planning algorithm while the other was conducted with the fixed reference gait. Torques of both hip and knee f/e joints were recorded during the whole walking tests. The combined CoP was calculated and recorded before each swing phase.

## 4. Results and Discussion

**Figure 11:**
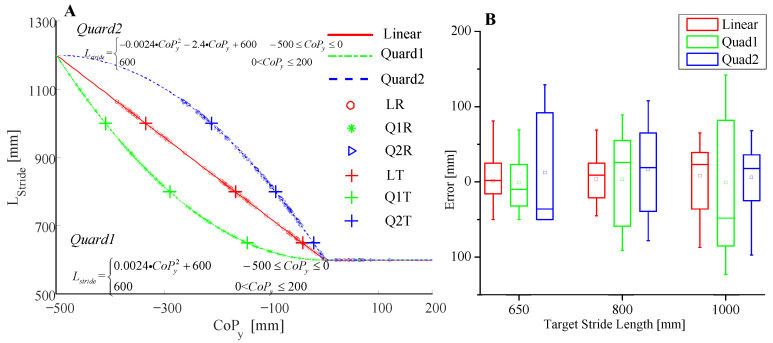
Stride length mapping function verification tests. (**A**) Walking test results with target stride lengths. “Linear” denotes the linear mapping function as shown in Equation (2), “Quad1” and “Quad2” are two quadratic functions for comparison. “LR”, “Q1R”, and “Q2R” are test results (the combined CoP and stride length) with the Linear, Quad1, and Quad2 mapping functions, respectively. “LT”, “Q1T”, and “Q2T” are the target stride lengths for the test with the Linear, Quad1, and Quad2 mapping functions, respectively. (**B**) Errors toward the target stride length of each test. For each mapping function of each target stride length, all 5 volunteers’ results were concerned.

The results of the first experiment are shown in Figure 11. In addition, two quadratic mapping functions were built for comparison, which are shown in Figure 11a. For each test, the mapping function determined the relationship between the combined CoP and stride length. The location of the resultant stride lengths in Figure 11a indicates that the linear mapping function helps the subject adjust to the target stride length easier. Figure 11b shows the stride length error of each test. For all three target stride length trials, errors with the linear function were smaller, which means that, with linear characteristics, the subjects could adjust their weight distribution easier to map the target stride length. Therefore, the linear mapping function was selected at this stage since it built a more transparent relationship between the combined CoP and stride length.

**Figure 12:**
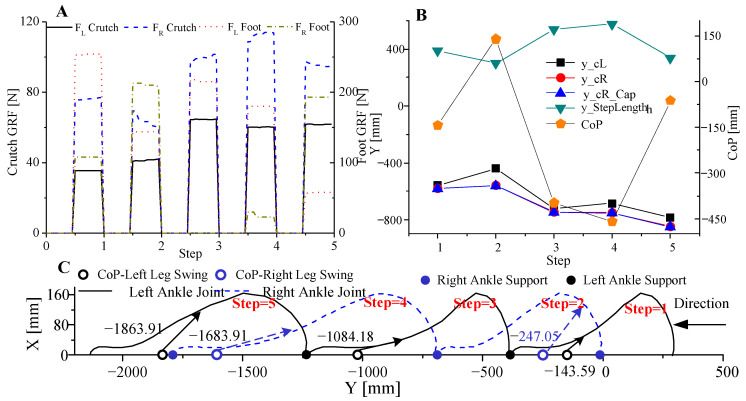
Intermediate variables and L/R ankle joint trajectories. (**A**) Sampled and preprocessed L/R crutches and exoskeleton sole GRFs before the swing phase, representing contributions for human-machine support. GRFs during the other phases were set to 0 here for clarity. (**B**) Calculation results of L/R crutch endpoint coordinates (y_cL and y_cR), step length, combined CoP coordinates, and right crutch endpoint coordinate from the motion capture system (y_cR_Cap), representing bases of the bgait planning algorithm. (**C**) L/R ankle joint Cartesian position coordinates and corresponding combined CoP (hollow circle) and support ankle joint (solid circle). The arrows indicate the corresponding relationship between CoP and ankle joint Cartesian position. Except for step 2, each CoP was outside L/R legs before the swing, and finally located within L/R legs after the swing as shown here.

Figure 12 shows resultant GRFs, the combined CoP locations, and trajectories of ankle position in the sagittal plane. Since the stride length is determined by the mapping function, Equation (2), at the end of the swing phase, the combined CoP (coordinate on *Y*-axis) is always located between L/R legs. The results in Figure 12c confirm this conclusion. Therefore, a balanced walking state is ensured at this stage and less strength is applied to the crutches at the moment. For the analysis of the online gait planning algorithm, intermediate variables sampled and processed before each step are shown in Figure 12a. $F_{L}$ Crutch and $F_{R}$ Crutch are the respective normal components of the L/R crutch GRFs. $F_{L}$ Foot and $F_{R}$ Foot are the respective exoskeletons L/R sole GRFs. During step 2, the L/R crutches-related components for CoP were lower because of a smaller forward-leaning range of the subject’s upper body, i.e., the subject was less dependent on the support of the crutches, leading to a positive CoP (see Figure 12b). According to the local coordinate system shown in Figure 1, this positive value represented that the combined CoP was between the L/R legs (blue hollow circle on the right side in Figure 12c). Thus, the corresponding stride length was set to a minimum value of 600 mm for smooth walking. According to steps 3 and 4, as shown in Figure 12a, the L/R crutch GRFs dominated, leading to negative values of CoP (see Figure 12b). Consequently, the combined CoPs were located ahead of the L/R legs (the black hollow circle near −1000 and the blue hollow circle near −1500 in Figure 12c).

**Figure 13:**
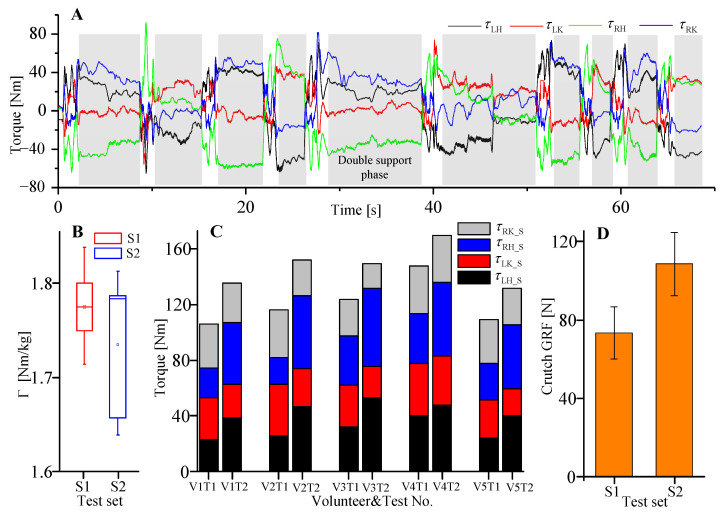
Experimental results of 2 walking test sets. (**A**) Driven torques of 4 joints during one 10-step walking trial. $\tau_{\mathrm{LH}}$, $\tau_{\mathrm{LK}}$, $\tau_{\mathrm{RH}}$, and $\tau_{\mathrm{RK}}$ are the torques of the left hip, left knee, right hip, and right knee, respectively. (**B**) Box plot of the normalized sum of 4 joint torques during the swing phase based on 5 volunteers’ trials. S1 and S2 denote test set 1 (walking trials with online gait planning based on the combined CoP) and test set 2 (walking trials with fixed reference gait), respectively. (**C**) Comparison of joint torques during the double support phase. $\tau_{\mathrm{LH}\_S}$, $\tau_{\mathrm{LK}\_S}$, $\tau_{\mathrm{RH}\_S}$, and $\tau_{\mathrm{RK}\_S}$ are torques of the left hip, left knee, right hip, and right knee during the double support phase, respectively. (**D**) Average L/R crutches GRFs measured after each swing phase. For each test set, L/R crutches GRFs of each step were measured and averaged based on 5 volunteers’ walking tests.

With the third experiment, the driven torques of the hip and knee f/e joints, as well as L/R crutch GRFs, were measured and compared between test set 1 (walking trials with online gait planning based on the combined CoP) and test set 2 (walking trials with fixed reference gait). Figure 13a shows the driven torques of 4 joints during 10 steps walking trial. The grey area selected parts to denote joint torques of the double support phase, and the remaining parts denote the joint torques of the swing phase. During the swing phase, the subject’s lower limbs are dragged or pulled to walk by exoskeleton joint torques, while during the double support phase, the subject’s lower limbs are maintained as a changeless posture with the help of exoskeleton joint torques. Thus, joint torques are generated within both phases for motion control. Figure 13b is a box plot of the normalized sum of four joint torques during the swing phase based on the five volunteers’ trials. The normalized torque is acquired with:

(15) $$\Gamma_{k}=(\sum_{i=1}^{10}\sum_{j=1}^{4}\tau_{kij})/(10m_{k}),$$

where $\Gamma_{k}$ denotes the normalized torque of the *k*th volunteer, *k* = 1, 2, … 5, $\tau_{kij}$ is the *j*th joint torque of the *i*th step for the *k*th volunteer, *j* = 1, 2, … 4, and *i* = 1, 2, … 10, $m_{k}$ is the weight of the *k*th volunteer. Normalized torques of S1 and S2 are closed, which indicates that the summed driven torques of four joints during the swing phase are almost the same between the walking test with the online gait planning algorithm and the walking test with the fixed reference gait. However, compared to the walking test with the fixed reference gait, the driven torques of four joints were reduced during the double support phase with the online gait planning algorithm (Figure 13c). The average reduction of joint torques for 5 volunteers is 18.5%. This reduction, on the one hand, may indicate that when the stride length is determined based on the combined CoP (to ensure that it is located between L/R legs), fewer torques are needed to maintain a double support posture. While, on the other hand, it may result from that the subjects using their own body forces and moments to maintain balance since the subjects were able-bodied volunteers. Furthermore, L/R crutches GRFs were measured after each swing phase. The average values of the two aforementioned test sets are shown in Figure 13d. There is an average reduction of 32.4% of crutch GRF when the online gait planning algorithm was applied. This mainly results from a better balance state of the human-machine system during the double support phase.

The present work mainly focuses on the validation and verification of an online gait planning algorithm based on the combined CoP of human-machine and crutch GRFs. The target of gait planning in this research is to gradually reduce the subject’s dependence on crutches for stability control. However, the relationship between the combined CoP and stride length has not been studied in detail. Our plans for future work will focus on behavior analysis of the SCI patient when walking with crutches, and further investigate the relationship between the combined CoP and stride length.

## 5. Conclusions

In this work, an online gait planning algorithm was presented, which is adaptive to combined CoP based on both human-machine and crutch GRFs. The mapping relationship between the combined CoP and stride length was preliminarily constructed with a linear function and validated by a comparison experiment. According to the experimental results, the algorithm can process the sensing signals and plan the landing point of the swing leg to ensure balance and stable walking. The performance of the gait planning algorithm was validated by the data analysis of the results, i.e., an 18.5% reduction of joint torques and a 32.4% reduction of crutch GRF during the double support phase compared to the fixed reference gait. This is of great significance for the further training of independent walking stability control in exoskeletons for SCI patients with less assistance of crutches. With this algorithm, the SCI patients can try to adjust their posture with a self-determined range by adjusting the weight distribution between the exoskeleton soles and crutches, because the corresponding gait with walking stability is planned accordingly. Thus, the SCI patient can be guided gradually by a physical therapist based on professional experience, or automatically by the exoskeleton system. The work in this paper forms the basis of a new solution for exoskeleton-assisted walking stability control with less help of crutches, i.e., gradually training the patient to be compatible with the exoskeleton for balanced walking. In the future work, the crutch model should be improved to consider it in the X-Y-Z coordinate, with which the real vertical component of GRF could be obtained instead of performing calibration. Meanwhile, paraplegic patients need to be tested to further verify the improvement of walking balance.

## Figures and Tables

**Table 1:** **Denavit-Hartenberg** (DH) parameters of the lower-limb exoskeleton.

*i*	$\alpha_{i-1}$	$a_{i-1}$	$d_{i}$	$\theta_{i}$
**1**	0	0	0	$\theta_{1}$
**2**	0	$L_{1}$	0	$\theta_{2}$
**3**	0	$L_{2}$	0	$\theta_{3}$
**4**	0	$L_{2}$	0	$\theta_{4}$
